# Intraoperative Laser Speckle Contrast Imaging For Real-Time Visualization of Cerebral Blood Flow in Cerebrovascular Surgery: Results From Pre-Clinical Studies

**DOI:** 10.1038/s41598-020-64492-5

**Published:** 2020-05-06

**Authors:** Antonella Mangraviti, Francesco Volpin, Jaepyeong Cha, Samantha I. Cunningham, Karan Raje, M. Jason Brooke, Henry Brem, Alessandro Olivi, Judy Huang, Betty M. Tyler, Abhishek Rege

**Affiliations:** 1 Department of Neurosurgery, Johns Hopkins University, Baltimore, MD United States; 2 Vasoptic Medical, Inc., Baltimore, MD United States; 3 Department of Oncology, Johns Hopkins University, Baltimore, MD United States; 4 Department of Ophthalmology, Johns Hopkins University, Baltimore, MD United States; 5 Department of Biomedical Engineering, Johns Hopkins University, Baltimore, MD United States; 6 Department of Neurosurgery, Catholic University School of Medicine, Rome, Italy

**Keywords:** Optical imaging, Medical research

## Abstract

Cerebrovascular surgery can benefit from an intraoperative system that conducts continuous monitoring of cerebral blood flow (CBF). Such a system must be handy, non-invasive, and directly integrated into the surgical workflow. None of the currently available techniques, considered alone, meets all these criteria. Here, we introduce the SurgeON™ system: a newly developed non-invasive modular tool which transmits high-resolution Laser Speckle Contrast Imaging (LSCI) directly onto the eyepiece of the surgical microscope. In preclinical rodent and rabbit models, we show that this system enabled the detection of acute perfusion changes as well as the recording of temporal response patterns and degrees of flow changes in various microvascular settings, such as middle cerebral artery occlusion, femoral artery clipping, and complete or incomplete cortical vessel cautery. During these procedures, a real-time visualization of vasculature and CBF was available in high spatial resolution through the eyepiece as a direct overlay on the live morphological view of the surgical field. Upon comparison with indocyanine green angiography videoangiography (ICG-VA) imaging, also operable via SurgeON, we found that direct-LSCI can produce greater information than ICG-VA and that continuous display of data is advantageous for performing immediate LSCI-guided adjustments in real time.

## Introduction

Intraoperative monitoring of cerebral blood flow (CBF) provides crucial information during neurosurgical procedures carried out for the management of intracranial aneurysm clipping, vascular bypass, and removal of arteriovenous malformations (AVM), among others^1^. When removing an AVM, CBF monitoring helps to identify the major draining veins and to detect the AVM nidus on the surface as well as the arterial inputs^2^. During revascularization procedures, optical visualization of the CBF can provide information regarding patency of bypass and entity of blood flow^3^. In aneurysm clipping, CBF monitoring helps to identify any modification of distal branch perfusion or any residual disease^4,5^. Perhaps most importantly, CBF monitoring can help avoid injury to perforating arteries, still the major cause of postoperative morbidity in cases of aneurysm, especially when the clipping is performed in locations such as the basilar bifurcation and anterior choroidal artery^6^.

Current intraoperative techniques for imaging vascular perfusion include digital subtraction angiography (DSA)^7,8^, indocyanine green angiography videoangiography (ICG-VA)^2,5^, dual-image videoangiography (DIVA)^9,10^, and fluorescein videoangiography (f-VA)^11^. These techniques are invasive because they rely on the injection of contrast agents into the body, may carry risk of vascular injury and stroke, or require the assistance of a neuroradiologist and the use of iodinated contrast and ionizing radiation^4,12–14^. These dye-based techniques can confirm success or failure of cerebrovascular surgery such as aneurysm obliteration and can allow for an assessment of anatomic results, of graft patency, or residual nidus in arteriovenous malformation (AVM)^7,15,16^; however, they are not able to provide real-time, continuous, and direct feedback on the CBF changes to the neurosurgeon during these procedures^17^. DSA remains the gold standard for the visualization of stenosis and/or residual disease^18^, despite the fact that it is not available in many institutions, has a higher rate of complications if not performed by well-trained personnel, and has no functionality for intraoperative detection of ischemic injuries or other abnormalities that could lead to irreversible neurological deficits or further surgical procedures^6,18–20^.

ICG-VA has become, in nearly all vascular neurosurgical procedures, a routine adjunct to confirming the viability of anastomoses and the patency of parent vessels. A recent alternative system is the DIVA, which requires conventional ICG injection but allows for a greater depth of field and the simultaneous visualization of both the light and the NIR fluorescence images of ICG-VA^9,10^. Intraoperative systems such as the Zeiss FLOW 800 (Carl Zeiss Meditec AG, Germany), Leica FL800 (Leica Microsystems, IL, USA), and SPY Elite (Stryker, MI, USA) include modules to analyze ICG-VA data to obtain additional information about the temporal distribution of the dye in the field of view during ICG-VA. The Zeiss FLOW 800 displays the ICG transition in pseudo-color and offers a detailed view of vessels suitable for bypass surgery as well as other proximal and distal vasculature^21^. However, the reliance of these instruments on dye administration restricts their use for conducting continuous or repeated quantitative assessments of blood flow in real-time. Assessment of flow is further confounded by analysis of the dye transient time which varies by location and speed of injection and is not sensitive to vessel diameter^22,23^. These techniques present major limitations. On the one hand, they are tied to the rate and dose of ICG dye injection and, on the other, they preclude the real time monitoring of the fluorescence and the quantitative analysis of the CBF^22^. Compared to ICG-VA, the f-VA can display vascular flow directly through the microscope oculars through a fluorescence filter module (YELLOW 560); however, f-VA is not paired with software to study the pattern of dye distribution through time and fluorescein takes longer to clear. It is also reportedly less sensitive to flow velocity during first pass^11^.

Direct quantitative flow measurements can be performed with microvascular Doppler ultrasonography (MDU)^12^ or optics-based Doppler probes, which measures volume flow and indicates the flow direction in donor/recipient vessels. However, insofar as it can measure the flow as well as the patency of the anastomosis, MDU aggregates flow under a probe tip with limited spatial resolution. Its placement can be difficult especially in a deep surgical field and, most importantly, will require additional and sometimes multiple manipulations of the target vessels^22,24,25^.

There is therefore a strong rationale for an easy-to-use, reliable, and high-resolution technique to enable intraoperative, direct, and simultaneous visualization of blood flow in multiple vessels within the surgical field in order to provide the neurosurgeon with real-time feedback of the CBF during the delicate and often irreversible surgical maneuvers of cerebrovascular surgery^6^. Laser Speckle Contrast Imaging (LSCI) is a non-invasive optical imaging method that has been utilized to obtain blood flow information in a range of applications including ophthalmology, dermatology, and neuroscience^26,27^. LSCI relies on image acquisition and processing of speckle patterns that are created when tissue is imaged under laser illumination. Orderly flow of blood cells inside vessels causes a blurring effect in the speckle pattern that can be quantified by assessing intensity fluctuations in the imaging data^28,29^. Typically, a quantity called speckle contrast is obtained at each pixel by computing the coefficient of variation of pixel intensities within its spatio-temporal neighborhood. Speckle contrast is related to the flow of the scattering particles and, therefore, this method is able to estimate a blood flow velocity index at every pixel, leading to the creation of blood flow imaging^28,29^. CBF monitoring via LSCI has already been tested on animal models and has recently been investigated for different clinical neurosurgical applications such as surgical revascularization^30^, awake functional mapping^31^, and cortical spreading depolarization after stroke^32–34^, as well as cerebral bypass and arterial grafting^13^. In these cases, however, LSCI imaging did not prevent interruption of the surgical workflow and did not allow for the direct visualization of the CBF which was only analyzed after the procedures.

To overcome these limitations, we developed a direct laser-speckle-video-imaging system, the SurgeON™ system, to capture and display blood flow information in real time. The SurgeON system, which we introduce here for the first time, complements the surgical microscope with real-time LSCI and works by displaying a low latency, on-demand video feed of vessel-specific blood flow information directly on the operator’s viewing eyepiece. In this work, we describe our preclinical experience and show how the system, which can be switched on and off as frequently as needed during the course of the surgery, is capable of supplying post-surgical CBF verification as well as instantaneous feedback and potentially continuous real-time CBF monitoring without latency and without diverting attention away from the working field during procedures. The SurgeON system also allows the surgeon to add real-time ICG videoangiography (ICG-VA) imaging to the structural-functional LSCI-based imaging displayed through the eyepiece. This enabled us to conduct a comparison of LSCI and ICG-VA modalities and to show that direct-LSCI imaging can provide more useful information at greater convenience than ICG-VA imaging. We demonstrate the preliminary proof of concept that, coupled with the neurosurgical benefits of this microscope integrated system, LSCI has the potential to become a routine tool for reliable and continuous intraoperative CBF monitoring during cerebrovascular surgery.

## Results

### Design and characterization of the LSCI system

**Figure 1:**
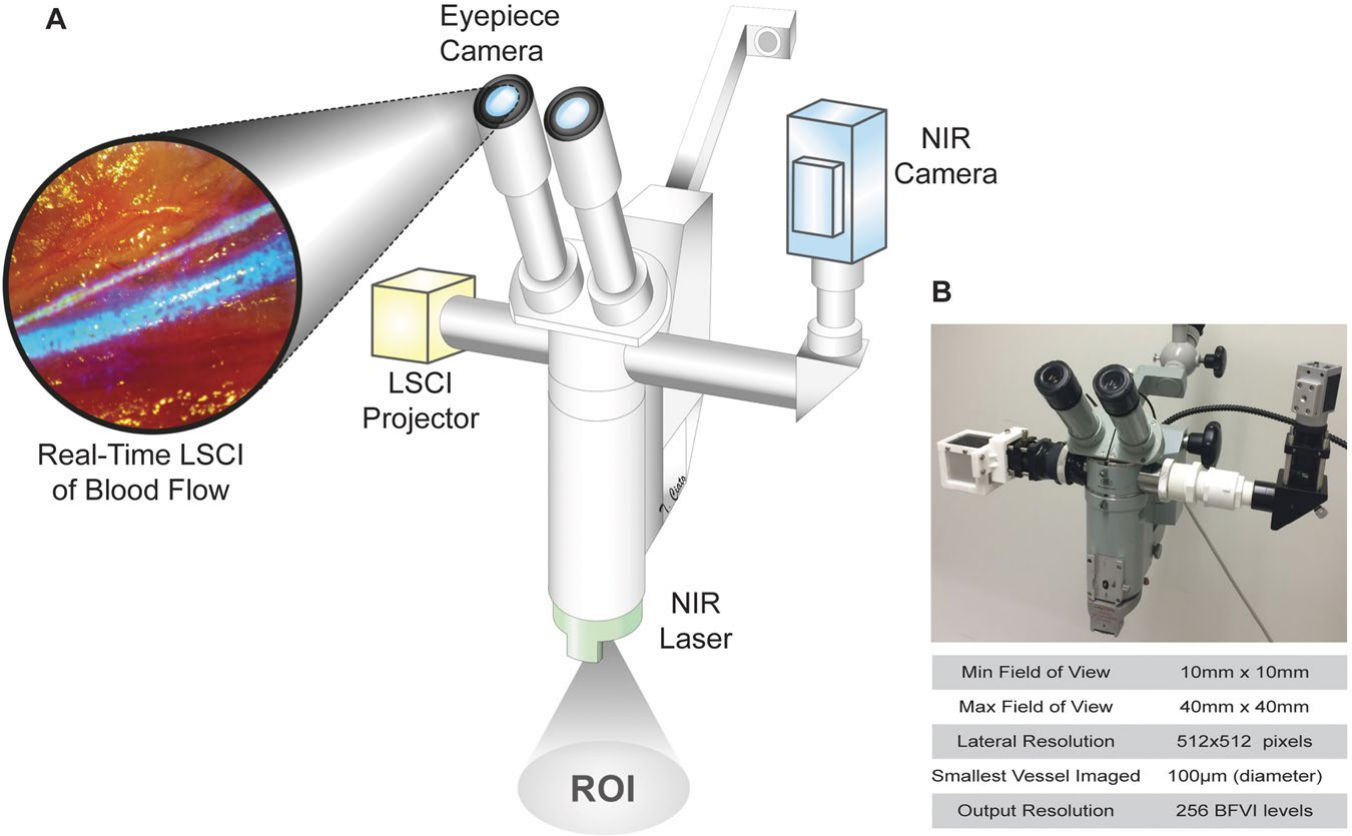
SurgeON System Schematic and Specifications. (**A**) As shown in the schematic, the SurgeON System comprises a surgical microscope modified to include the following key components: a near infrared (NIR) laser source (green) irradiates the target ROI images which are then captured by the NIR camera (blue). This camera is connected to a PC via MATLAB environment where these acquired laser speckle data are processed in real-time and a video-feed of the resulting blood flow information is transferred to the LSCI projector (yellow) to be seen by the operator through the microscope eyepiece. (**B**) The SurgeON System has imaging specifications that are suitable for neurosurgery.

Intraoperative direct CBF monitoring and LSCI was developed using the newly designed direct LSCI Imaging, the SurgeON System, as shown in Fig. 1. A Carl Zeiss OPMI surgical microscope was used as the optical backbone; one light source was replaced with a near infrared (NIR) laser source (peak: 830 nm), while an external broadband light source was added to compensate for decreased illumination. A high-speed NIR CMOS camera was also fitted onto the microscope’s viewport to capture images of the target with the same magnification and focus as seen by the operator. A narrow-band NIR filter (peak 830 nm) was employed to receive light from either the laser illumination or the fluorescent emission of indocyanine green, in order to perform both LSCI and ICG-VA. Illumination for ICG-VA was provided by an external laser with peak wavelength of 780 nm. The camera was connected to a desktop PC via MATLAB (Mathworks, MA), where the acquired laser speckle data were processed in real-time to provide an output in HDMI video format to a projection module. Through a modified beam-splitter arrangement, this video feed was projected into the eyepiece as an overlay on the target field of view.

To achieve real-time processing of laser speckle images, a scheme called spatio-temporal LSCI (or *stLSCI*) was used to generate blood flow imaging by estimating the blood flow velocity index (BFVI) at every pixel, as is illustrated in Supplementary Fig. 1. When operational, the SurgeON system was able to produce BFVI images with a lateral resolution of 512 × 512 pixels and a frame rate that was consistently greater than 60 Hz, and a latency between input and output images of less than 50 milliseconds. The magnification and focusing function of the surgical microscope permitted visualization of blood flow within a circular field of view with a diameter ranging from 10 mm to 40 mm. When the microscope is set to its largest magnification setting, each camera pixel corresponds to 19.2 µm in the object plane (surgical field). Since the SurgeON system uses a 5 pixel × 5 pixel window in the spatial domain for speckle contrast calculation, information is aggregated from ~100 µm square region in the surgical field. However, because this window is sliding, this produces a smoothing effect rather than compromising the spatial resolution resulting in the ability of the SurgeON system to resolve flow in vessels of diameter 100μm. Before proceeding to *in vivo* experimentation, we tested the sensitivity and resolution of the SurgeON System using an *in vitro* microfluidic system (Supplementary Fig. 2)

### Real-time tracking of *invivo* blood flow changes

To assess the efficacy of this system we tested it first *in vivo* by imaging femoral vessels in five rats. Under SurgeON, we performed LSCI to monitor blood flow changes in the femoral vessels under basal conditions, during temporary clipping of the artery, and during reperfusion after removal of the clip. During each of the three conditions, five imaging sessions were performed to assess the *in vivo* inter-session reproducibility of the measured blood flow. Because blood flow in the femoral artery and vein is inherently pulsatile, the inter-session reproducibility for *in vivo* BFVI measurements was determined using mean BFVI values in each vessel over the entire imaging session comprising at least 4 cardiac cycles. The inter-session coefficient of variation (CV) of BFVI measurements across all animals was determined to be 5.91% ± 7.33% for the femoral artery, and 1.43% ± 0.56% for the femoral vein.

**Figure 2:**
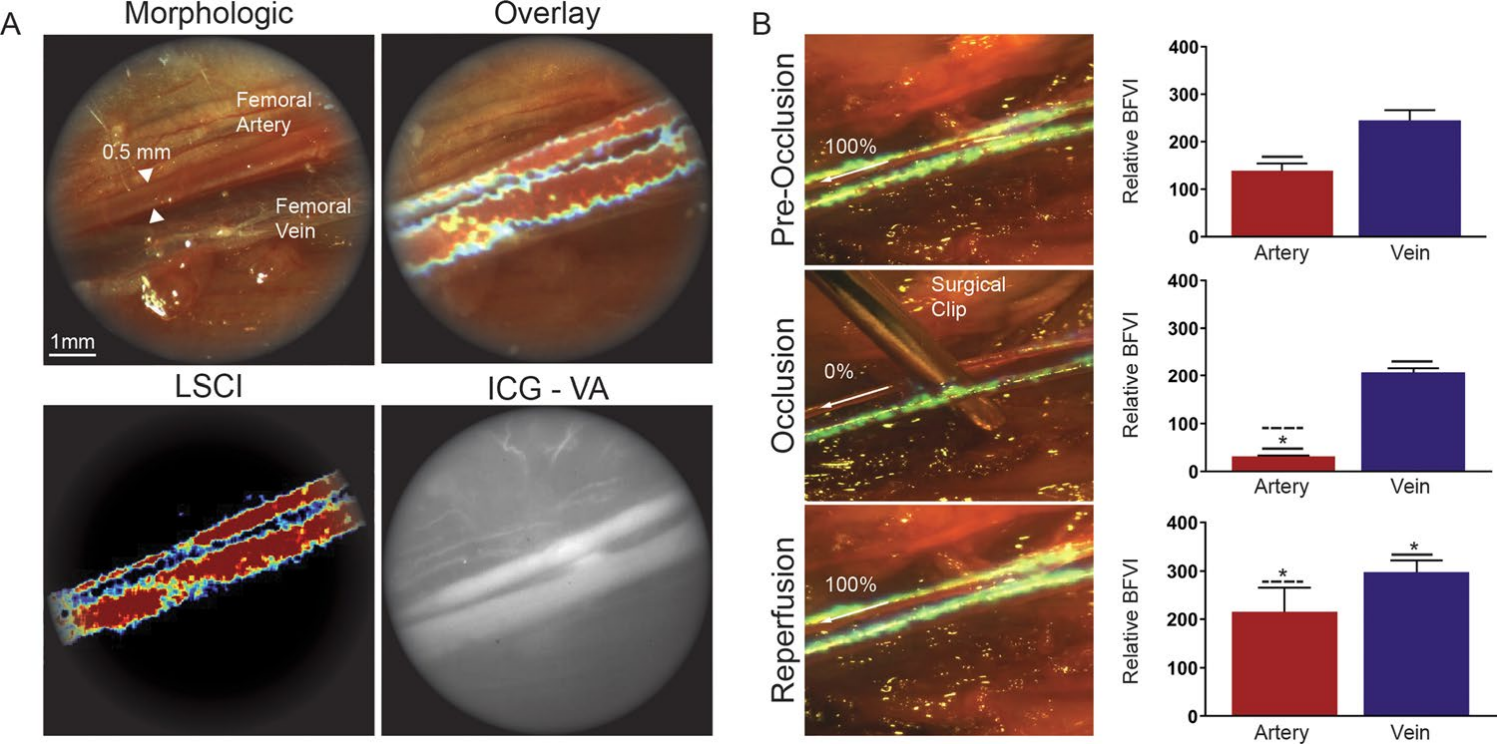
SurgeON shows *in vivo* blood flow changes in real-time. (**A**) Frames of the recorded video during real-time visualization of the femoral artery and vein through the microscope’s eyepiece using morphologic, LSCI, LSCI overlay on morphological images and ICG-VA. (**B**) Frame of the real-time LSCI through the eyepiece microscope during the femoral artery clipping procedure. The bar graphs show the Mean + /- SE of the relative blood flow velocity indices (1/tauc values) acquired in 5 sets per each condition in 5 different rats in separate experiments.

The microscope eyepiece allowed us to gather both data and real-time images in four different modes: pure morphologic, LSCI (BFVI), morphologic with overlaid LSCI (BFVI), and ICG-VA (Fig. 2A). The clip placement on the femoral artery caused a decrease of the LSCI signal which corresponded to a significant reduction of the measured BFVI values distal to the clip (p < 0.0001). Similarly, the clip removal restored the LSCI signal with a corresponding significant increase of the measured BFVI values (p < 0.0001) (Fig. 2B). Immediately following the removal of the clip, the reperfusion BFVI value was significantly higher than the basal condition (p = 0.0009); however, this difference was not distinguishable on LSCI and was assessed via retrospective analysis. The clip placement on the femoral artery did not cause a significant decrease in the BFVI values in the femoral vein; however, release of femoral artery occlusion resulted in a significant increase in the venous BFVI values (p < 0.0001).

### Continuously available display of cerebral blood flow information

**Figure 3:**
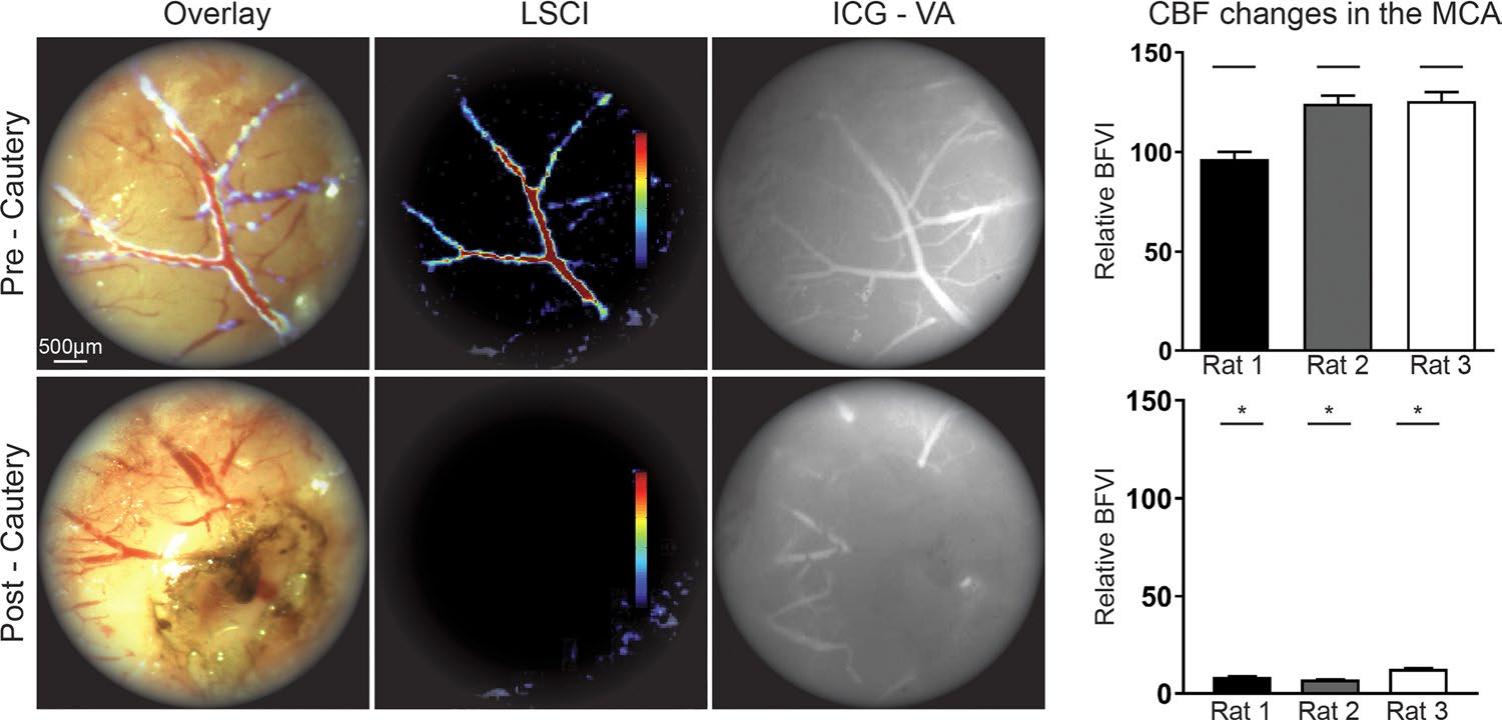
SurgeON tracks CBF changes during MCA cautery. Frames of the real-time MCA images through the microscope’s eyepiece: morphologic view with overlaid LSCI, LSCI and ICG-VA images. Bar graphs showing significant (p ≤ 0.0001) decrease in blood flow velocity indices estimated using laser speckle contrast imaging after vessel cautery in each rat.

**Figure 4:**
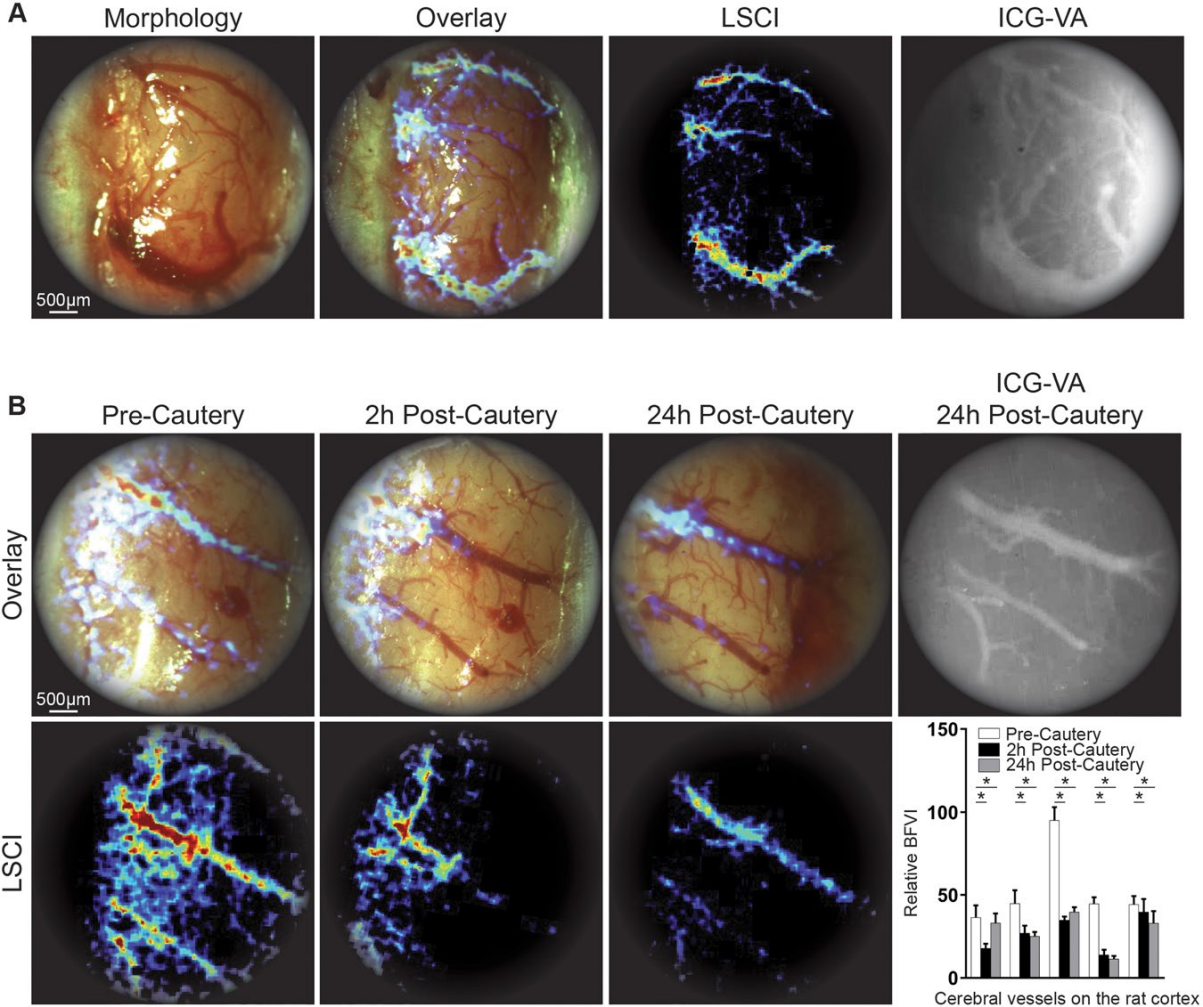
SurgeON provides more information than ICG-VA on CBF variation following MCAO. (**A**) Frames of real-time visualization of the cortical vessels through the microscope’s eyepiece: morphologic, LSCI, overlay of morphologic and LSCI and ICG-VA for comparison. (**B**) Representative real-time morphologic images with overlaid LSCI and LSCI only during pre-cautery, 2 hours post-cautery, 24 hours post-cautery and ICG-VA at 24 hours showing CBF decrease at 2 hours compared to the basal CBF and a partial restored blood flow at 24 hours. Bar graph showing mean + /−SE of blood flow velocity indices measured and aggregated across three vessels in each rat brain, in a total of five rats that underwent this procedure as part of three separate experiments. Note that ICG-VA reveals lingering fluorescence even 24 hours post cautery, possibly exacerbated by lack of clearance in a flow-arrested vessel whereas the noninvasive, quantitative LSCI technique clearly demonstrates a CBF reduction after MCAO.

To assess the capability of this system to image vessels within the brain parenchyma during a cerebrovascular procedure, we performed middle cerebral artery (MCA) cautery in a rat model. During the procedure, the surgeons used the microscope eyepiece to obtain real-time data and to directly visualize the MCA blood flow in basal condition (pre-cautery) and after cauterization (post-cautery) using the following imaging modes: pure morphologic (not shown), LSCI (BFVI), morphologic with overlaid LSCI (BFVI), and ICG-VA. After cautery, LSCI clearly revealed a significant reduction in the blood flow within the MCA as shown in the image captured by the eyepiece camera, as expected and confirmed by offline post analysis of BFVI measurements made in the imaged branch of the MCA (p < 0.0001) (Fig. 3). Next, to assess the efficacy and sensitivity of the LSCI system on the monitoring of CBF changes in small cortical vessels, we imaged the rat brain cortex through a left fronto-parietal craniectomy after occlusion of the ipsilateral MCA (MCAO) once again using both LSCI and ICG-VA modalities (Fig. 4A). Data analysis was conducted for the BFVI values in the cortical vessels under basal conditions (pre-MCA cautery), at two hours after, and at twenty-four hours after MCA cauterization (2 hours post-cautery and 24 hours post-cautery). At two hours post MCA cautery, LSCI revealed a dramatic decrease in CBF in the cortical vessels. This was shown in real-time directly through the eyepiece of the surgical microscope and was later confirmed by offline post-analysis of BFVI measurements. At 24 hours post-cautery, LSCI showed that, compared to two hours post-cautery, some blood flow returned to the imaged vessels possibly due to blood recruited from the anastomosis; however, the BFVI values, as observed in real-time from their pseudo-color representation, and confirmed through post-analysis of recorded BFVI measurements, was significantly lower than in basal conditions. At the same time point, unlike the LSCI data, the ICG-VA imaging of the brain cortical vessels provided no information on the reduced CBF following MCAO and was therefore unable to confirm the presence of CBF along those vessels (Fig. 4B).

**Figure 5:**
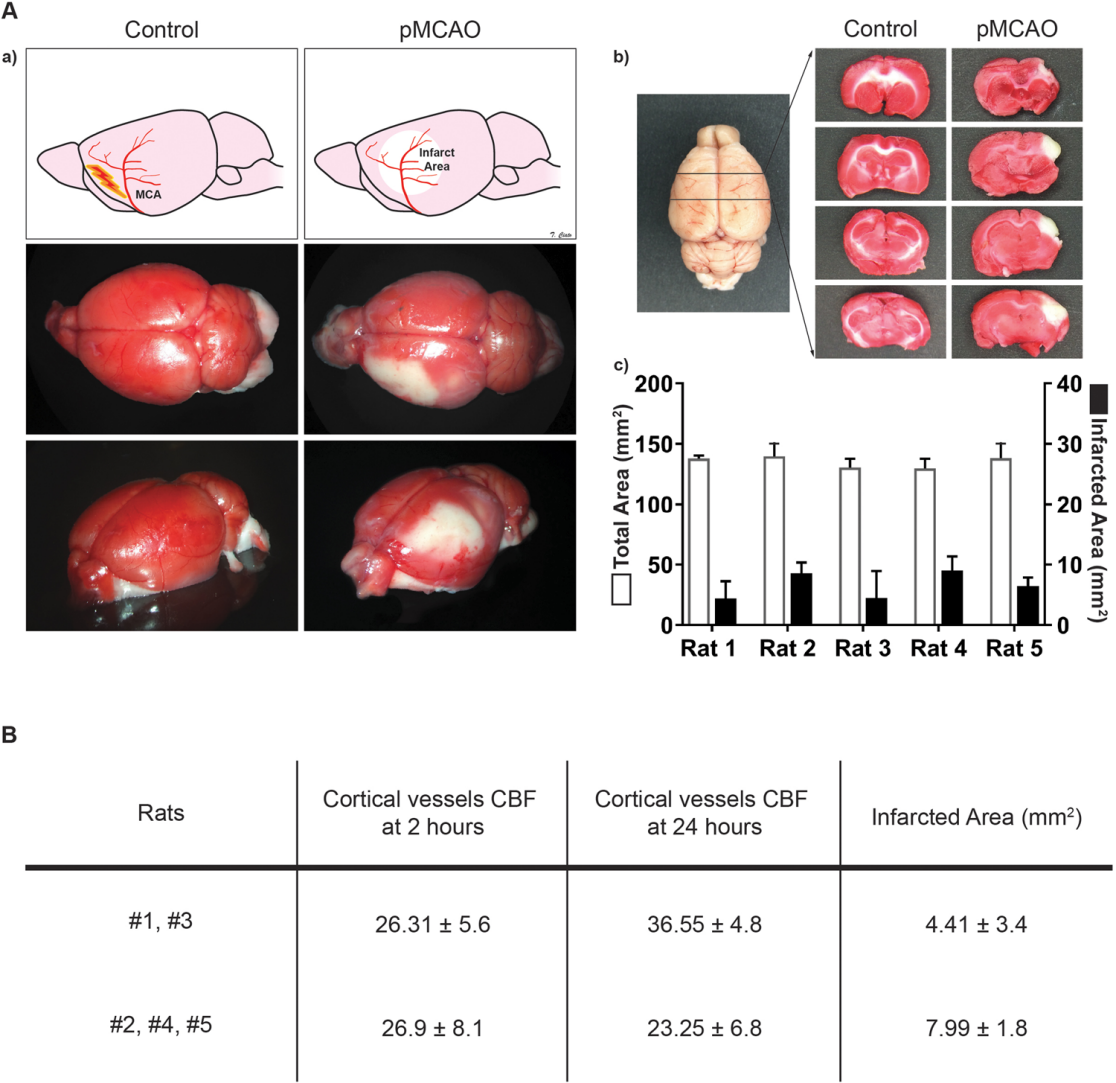
Cortical CBF changes following MCAO resulted in proportional infarcted brain tissue. (**A**) Schematic representation of the MCAO procedure and brain area analyzed by TCC. (a) Illustration and pictures of the brain analyzed, the brains were harvested from wild type control rats and from the rats that underwent MCAO 24 hours post-surgery, and all were treated with TCC staining. (b) The brains were sliced and the infarcted area (white area) was measured via ImageJ. (c) Bar graphs show for each rat the mean + /- SE of the total and ischemic areas of the all sectioned slices per brain (white and black, respectively). (**B**) The table shows that the CBF (1/tauc) values in the cortical vessel of the analyzed area 24 hours post MCAO correlate with the extent of the infarcted area, which tends to be bigger in the groups of rats with a lower CBF at 24 hours.

Moreover, to investigate the relationship between CBF changes in the cortical vessels and the resulting brain ischemic injury, the same rats were euthanized at 24 hours after imaging and the brains harvested for histological analysis of the size of the ischemic regions and compared with the LSCI-based CBF recordings obtained previously (Fig. 5A). In two of the five rats (#1 and #3), the CBF in cortical vessels was higher at 24 hours post-cautery than at 2 hours post-cautery, probably due to reperfusion phenomena, which may also explain the smaller area of infarct measured in these rats at 24 hours (Supplementary Table 1). In the remainder of three rats (#2, #4, and #5) the CBF in cortical vessels at 2 hours post-cautery and at 24 hours post-cautery was comparable without any significant increase, and concomitantly the size of the infarcts in this group was significantly larger (p < 0.0001) (Fig. 5B).

### Facilitation of intra-operative guided maneuvers during vascular surgery based on real-time blood flow changes

**Figure 6:**
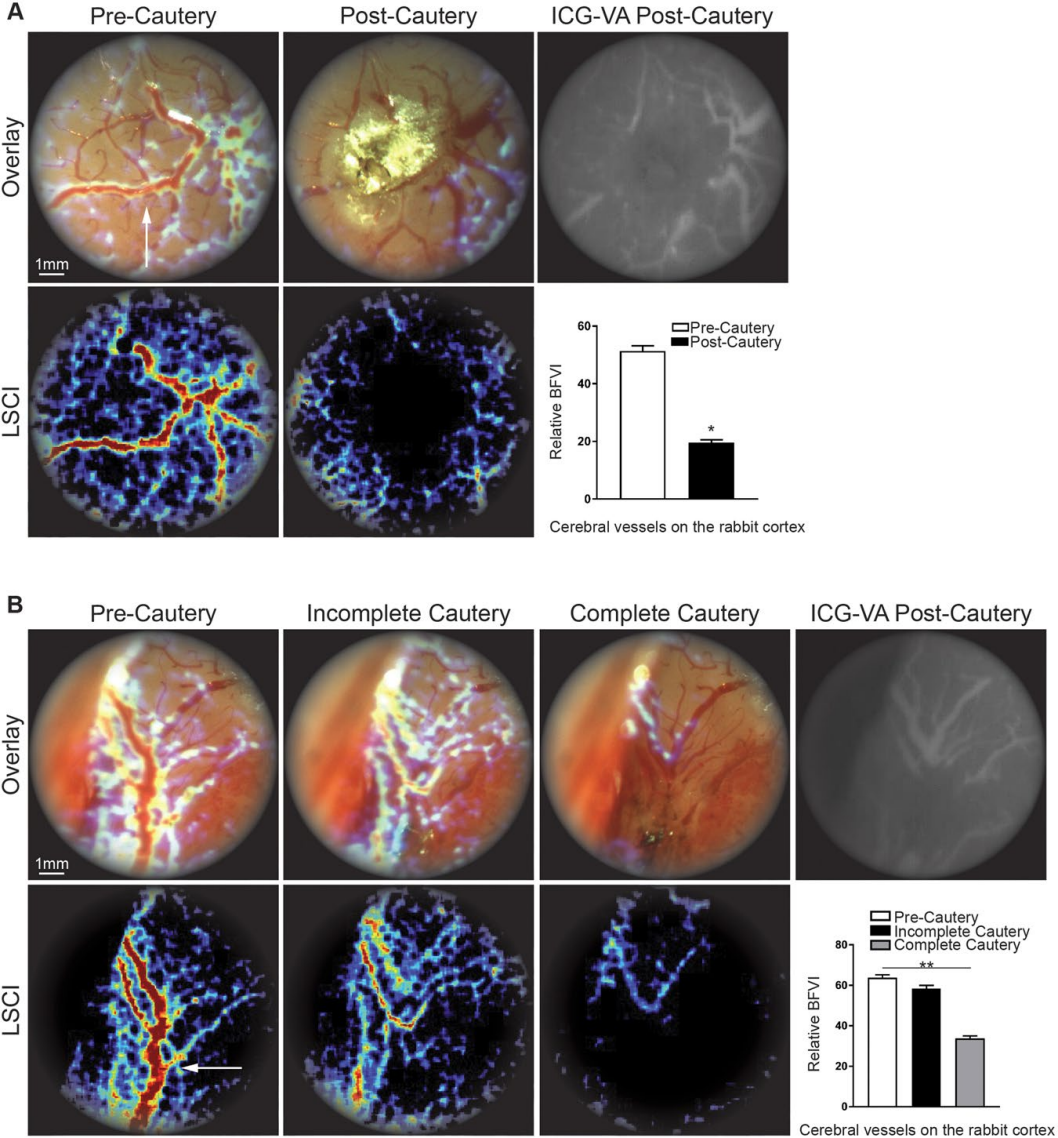
SurgeON detects in real time incomplete vessel occlusion and guides immediate adjustments during a vascular procedure. (**A**) Frames of the recorded real-time imaging of the rabbit cortical vessels through the microscope’s eyepiece: morphologic, LSCI, overlay of morphologic and LSCI and ICG-VA. (**B**) Real-time overlay and LSCI of the surgical field of view after the first unsuccessful vessel’s cautery attempt, and then after the second when upon LSCI guidance we performed a complete vessel cautery. The bar graph shows the significant CBF reduction obtained only after the complete vessel cautery. ICG-VA imaging confirms the absence of flow in the brain trunk of the target vessel however gives no information on the reduced CBF in the vessel branches.

To evaluate the advantages of intraoperative LSCI through the microscope during neurovascular surgery, and to investigate the performance of the SurgeON System on a different surgical target and over a larger field of view, the system was used to visualize CBF changes in a rabbit model. The procedure involved a left frontal craniotomy and, after identifying the vessel of interest, a brain cortical vessel cautery. LSCI displayed in real time the interruption of the CBF in the target vessel, as confirmed later via ICG-VA imaging upon ICG injection and also confirmed by the BFVI values measured via LSCI. (Fig. 6A) We subsequently repeated this maneuver in another vessel using a lower bipolar intensity. This time, when we switched the visualization of the FOV from basal morphological imaging to LSCI, we observed that the cautery performed was incomplete, indicating that the procedure had failed to cauterize and stop blood flow in the vessel of interest. We therefore proceeded to re-cauterize the vessel under real-time LSCI-imaging guidance by progressively raising the bipolar intensity until LSCI imaging signaled the cautery was complete.

Retrospective analysis of the recorded BFVI values confirmed the sequence of CBF changes observed in real time in the microscope eyepiece via the LSCI modality during the procedure, i.e., a decrease of the LSCI-based flow signal (which turned from a thick red visualization to thin light blue visualization) after the first cautery attempt (incomplete cautery), followed by a total absence of LSCI signal upon cautery completion. These CBF changes were not detectable at the microscope using the basal morphological view. At the end of the maneuvers described above, ICG-VA confirmed the completeness of the vessel cauterization corroborating the ability of the LSCI. To note, the ICG-VA imaging failed to show any CBF decrease in the branches of the cauterized cortical vessel probably as these branches were still patent and could receive ICG from other vessel branches (Fig. 6B).

## Discussion

Optical CBF imaging can play a crucial role in cerebrovascular procedures^35–37^. This study introduces a superior technical solution for noninvasive imaging and real-time monitoring of CBF and presents preliminary evidence of its advantages for intraoperative use. Unlike other imaging methods, the SurgeON system can display the CBF in pseudo-color directly within the eyepiece of the surgical microscope; it does not require any extra personnel in the OR nor the injection of any contrast or dye; and it can be used *on demand* as many times as needed during the course of surgery. Unlike the dye-based Flow 800 system, SurgeON can provide continuous real-time visualization of the CBF and its hemodynamic parameters, and can therefore be used for quantitative blood flow assessment^21,23^. Unlike ICG-VA, it dispenses with the delays associated with dye administration or clearance time. Unlike Doppler-based techniques, the spatial resolution of LSCI in conjunction with its seamless integration into a surgical microscope, makes it possible for LSCI to image multiple and differently-sized vessels simultaneously in the same FOV. Unlike DSA and microvascular Doppler flow probe, the use of this system can be performed without interrupting surgery and without exposing patients to additional surgical time and surgical maneuvers^22,23^.

The SurgeON system has proven effective and reliable in detecting CBF changes across different preclinical models and in surgical fields with multiple vessels, each with some relevance to clinical scenarios. In all cases, observation and recording was simply performed by switching view modes on the eyepiece of the microscope. CBF changes were not only displayed in pseudo-color aligned and overlaid on the surgical field of view, but also recorded for quantitative post-analysis. All procedures, such as femoral vessel clipping and brain cortical vessel cautery, were performed successfully under the microscope, where we monitored the vessels of interest in LSCI view and verified them as needed in overlay with the morphological view. Unless otherwise specified, each animal model of imaging blood flow variation in the brain had a sample size of five. Though the study was carried out on limited sample sizes, results were statistically significant as the differences of vascular flow between baseline and an occluded or obliterated state are profound. Additional animals would have been imaged if the statistical assessment had yielded inconclusive results.

We were also able to compare LSCI and ICG-VA side by side simply by switching the SurgeON System to ICG-view mode after dye injection. ICG-VA, besides requiring the use of a safe dye, is an otherwise practical and low-cost intraoperative technique with high spatial and temporal resolution^38^. However, though widely used in neurovascular surgery to verify vascular patency, this technique is liable to give false positive findings due to incomplete clearance of the dye from the vessels and, most importantly, due to the difficulties involved in interpreting ICG’s different patterns of brightness. A discerning eye and much experience is required to avoid misdiagnosing the retrograde filling of the branches distal to the aneurysm, to identify incompletely clipped aneurysms, and to distinguish between the residual ICG filling of an aneurysm and the diffuse weak fluorescence of thrombus, atherosclerosis, or calcification^14,15^. Previous studies have suggested that LSCI can outperform ICG-VA in showing both full and partial occlusion in terms of relative flow magnitudes^39,40^. In this study, where both techniques were sequentially but near-simultaneously used in the same preclinical settings and in the same surgical microscope, we confirmed those findings and demonstrated the overall superior profile of LSCI over ICG-VA. While LSCI successfully guided the operator in real time during vessel cautery surgery, ICG-VA only allowed for post-facto vessel patency verification. Furthermore, while LSCI was able to show CBF variation in the branches of the vessel, ICG-VA only provided binary flow/no-flow information of the cauterized vessel^40^. These differences can be of crucial importance to the outcome of surgery. Through improved discrimination of flow rates, the SurgeON system, unlike ICG-VA^41,42^, may enable detection of a partial aneurysm clipping or an imperfect bypass as they happen and guide immediate adjustments in real time, or possibly assist the surgeon with flow-based information when temporary clips are placed on significant vessels prior to permanent clipping or during bypass procedures. However, any of these applications and potential benefits of the SurgeON system remains to be validated clinically.

Depending on the type of analysis, LSCI is capable of providing information pertaining to two parameters – blood flow (blood volume per unit time in the vasculature) and blood perfusion (blood volume per volume tissue, per unit time) – that are distinct but yet related via the number and caliber of vessels in the region of interest^43^. We have seen that low blood flow corresponds to a lower perfusion and a wider infarcted area in the brain after MCAO; however, the relationship between these two variables needs to be analyzed with information on vessel density and diameters on a case-by-case basis^44,45^. There is still much debate as to the quantitative relationship with *absolute* blood flow, velocity, or tissue perfusion in LSCI^46^. There is no calibration of the inverse relationship between absolute flow velocity and the correlation time of speckle intensity fluctuations across subjects, tissues, models, and systems, and therefore its quantification lacks an agreed absolute value^46,47^. LSCI-based measurements have therefore been reported in the form of an index, BFVI, which will require further verification in patients in order to define the exact value of the proportionality constants^43^. However, in addition to our characterization of intra- and inter-session reproducibility of BFVI measurements, there is substantial validation of the LSCI technique to produce consistent measurements of BFVI in the same subject, as well as to monitor changes in longitudinal experiments^27,48^.

LSCI, like most optical techniques, requires line of sight access to the region being assessed, and may be susceptible to inaccuracies because of instruments in the field of view or motion artifact that may be unavoidable during some surgeries. LSCI is a two-dimensional technique and therefore images blood flow velocity components in plane and within the depth of field, which in our experiments was defined by the optical setup of the surgical microscope. While BFVI values relative to baseline remain unaffected, it is further recommended that LSCI be used to assess flow only in vessel regions that are in focus. Further, because LSCI does not track individual red blood cells but rather derives flow information from the bulk phenomenon of speckle blurring, percent hematocrit in the blood may potentially influence measurements^49,50^. However, this effect is not expected to be significant as long as there is no sharp decrease of hematocrit that breaks the continuum of particulate flow inside the vessels. In either case, BFVI values normalized to baseline should be robust to varying degrees of hematocrit. Finally, LSCI is susceptible to artifact arising from gross tissue motion, which presents as noise that may affect the sensitivity with which blood flow is discriminated in smaller vessels; however, this problem is addressable using *a priori* information about vessels and a mathematical model of gross motion that can be available through a set of initialization data^51^. During the *in vivo* experiments reported here, the real-time view provided by the SurgeON system was optimized to suppress noise from gross tissue motion by comparing BFVI values to preset thresholds linked to said artifact. However, no such thresholds were applied to the data that was recorded and analyzed offline.

These limitations are worth noting, in that they may be overcome through the implementation of real-time or offline noise detection schemes. Some of these are already under investigation in patients. A simple filter based on camera acquisition times has been shown to remove cardiac and respiratory motion noise and improve the quantitative flowmetry capabilities of LSCI. BFVI values have recently been remodeled and rescaled in a way that enables quantitative *in vivo* blood flow independently of hematocrit, blood volume, and RBCs changes from macro to microcirculation vessels^27,43,52^. Further research in humans may help to fine-tune the offset correction further, to the point where quantitative CBF monitoring, when conducted in microcirculation beds, will be unaffected by the size and density of the vessels outside the focal area. Moreover, it will be worthwhile, in future studies, to compare the SurgeON system with other intraoperative CBF measurement tools, such as the microvascular Doppler Flow Probe.

Despite the aforementioned limitations, the LSCI-based SurgeON System represents a uniquely suitable candidate for seamless integration into the neurosurgical workflow, which should provide the neurosurgeon with conveniently acquired, robust information pertaining to blood flow in the surgical field of view that may be practically useful for comparative assessment and dye-free confirmation of a vessel’s perfusion status.

Although further validation and research is needed for clinical translation and to test wider clinical applicability, this study demonstrates preliminary feasibility that this new technique was successfully able to monitor CBF changes using LSCI modes directly through the eyepiece of a microsurgical microscope, and could do so in real time and with optional overlay of morphological images, without interrupting the surgical procedure or losing sight of the morphological visualization of the surgical field. This system is non-invasive, quantitative, continuous, and high-resolution. Upon further clinical validation, it has the potential to be introduced into the surgical suite as a new routine intraoperative optical imaging technique to assist the neurosurgeon *in the* intraoperative management of cerebrovascular surgery, facilitate the learning curve of young neurosurgeons and, most of all, improve patient outcomes. Furthermore, due to its ability to provide real-time feedback on CBF and enable direct comparison between LSCI information and electrocortical stimulation, the SurgeON system promises to be of high scientific value in view of increasing interest in LSCI applications, such as in functional mapping or stroke research.

## Methods

### Theory and design

The SurgeON System relies on LSCI for estimation of blood flow information without the introduction of any dyes. As illustrated in Fig. 1, a prototype SurgeON system was developed by modifying an old surgical microscope (OPMI, Carl Zeiss). A laser module emitting light in the near infrared (NIR) spectrum (peak wavelength of 830 nm), was used to illuminate the region of interest for LSCI. A continuous stream of raw laser speckle images was acquired at a frame rate of 120 frames/second by a high-speed camera module mounted on one of the video ports. The camera module is comprised of a custom lens assembly for optimal image transmission, an aperture that determines the speckle size, and a bandpass filter (selective for NIR wavelengths) for increasing the signal-to-noise ratio in the acquired images. The stream of sequentially acquired images were processed in real-time on a GPU-based computer using MATLAB (Mathworks, MA) to generate a stream of output images wherein blood flow values were depicted in pseudo-color and transmitted to a projection module mounted on the second video port. The projection module was comprised of a projector (RIF6, NY) and a custom optical arrangement for adjusting magnification, alignment, and focus. The beam splitter that enabled this video port was reversed such that the ipsilateral eye piece could visualize the original field of view with a simultaneous overlay of dynamic blood flow images from the projection module.

### Imaging analysis and system set-up

LSCI provides a measure for blood flow velocity by quantifying the extent of *blurring* of dynamic speckles caused by the motion of red blood cells through the vessels. For each stack of acquired speckle image frames, the speckle contrast (K), defined as the ratio of standard deviation of pixel intensities to the mean pixel intensity within a spatio-temporal neighborhood of pixels around every pixel *P*_0_ (Eq. 1), was computed.

1 $$K({P}_{0})={\sigma }_{N({P}_{0})}/{\mu }_{N({P}_{0})}$$

where ${\sigma }_{{\rm{N}}({P}_{0})}$ and ${\mu }_{{\rm{N}}({P}_{0})}$ are the standard deviation and mean, respectively, in the intensity of all pixels on a defined local neighborhood N(*P*_0_). Traditionally, *K(P*_0_) values are calculated such that N(*P*_0_) is chosen exclusively in a single image frame^53^, or exclusively in the temporal domain for each pixel location^54^. However, to optimize the spatio-temporal resolution and image acquisition times, the SurgeON System calculates speckle contrast using a spatio-temporal pixel-neighborhood of 5 pixels × 5 pixels (spatial window, *S*) × 5 frames (temporal window, *N*) around every pixel *P*_0_ for contrast calculation^55^. The correlation time of intensity fluctuations observed in speckle dynamics over the exposure time of the camera, is known to be inversely proportional to the velocity of the moving scatterers, therefore can be used to estimate a blood flow velocity index (BFVI) at the location. Specifically, BFVI can be mathematically computed from *K* at every pixel at a given exposure time *T* of the camera using Eq. 2^56,57^.

2 $${K}^{2}=\frac{1}{T\times BFVI}\left\{2-\frac{[1-\exp (\,-\,2T\times BFVI)]}{T\times BFVI}\right\}$$

BFVI values computed at each pixel were compared against a preset threshold to remove background noise resulting from non-vascular and capillary flow. BFVI values in the higher-than-threshold flow regions were depicted in pseudo-color and overlaid on the original field of view (FOV) for the operator to visualize within the eyepiece. The five-frame image stack used for *stLSCI* computations is refreshed with every acquired laser speckle image on a first in, first out basis producing BFVI images at the speed of image acquisition.

Spatial and temporal performance characteristics were measured by imaging a standard grid and a high-speed timer, respectively, and analyzing the images captured by the SurgeON System camera and overlays captured by an eyepiece color camera module (ECCM). Note that the ECCM records the compound visualization of the FOV overlaid with BFV information, as would be available to the surgeon-operator. Images from the ECCM were recorded on a separate computer since this module was used only for characterization and validation, and is not fundamentally a component of the SurgeON System.

To calibrate our system, we used a syringe pump to infuse blood in a Tygon tube at multiple preset velocities and imaged the tube to calibrate the SurgeON System prototype (Supplementary Fig. 2A). Reproducibility of blood flow measurements made by the SurgeON System prototype was evaluated by calculating the CV in measurements at each of nine preset blood flow rates and at each of eight different exposure times of the camera. Exposure time was specifically chosen as an imaging parameter of interest because it determines the sensitivity with which blood flow rates can be discriminated, as evident from the calibration plots in Supplementary Fig. 2B. The mean CV was determined to be 2.00% ± 1.07%, and the blood flow calculated by the system and the actual blood flow measured with the syringe pump were determined to be statistically comparable with the coefficient of determination (R^2^) greater than 0.90 (p < 0.02).

### Characterization of imaging performance

Further, an additional laser source (peak wavelength: 780 nm) was added to the experimental setup to enable the SurgeON System to also perform IndoCyanine Green Videoangiography (ICGV), a common angiographic technique used for corroboration of perfusion status during surgery. The functional performance of the SurgeON System prototype was characterized using static and dynamic means. Static means involved imaging of standard grids to calculate minimum and maximum field of view, lateral resolution, latency, and rate of displaying blood flow information.

### Surgical procedures

#### Animals

Female F344 rats, weighing 125–175 g each (Harlan Bioproducts, Indiana, IN) and male New Zealand white rabbits were housed in standard facilities and were provided with ad libitum access to food and water. Experimental protocols were approved by the Johns Hopkins University Institutional Animal Care and Use Committee (IACUC) and all policies and guidelines were strictly followed throughout the studies. Unless otherwise specified, temporary/permanent occlusion of the femoral artery and of the brain cortical vessel was performed in five F344 rats. Five sets of BFVI measurements were made using the SurgeON System prototype and the ECCM at three stages—pre-occlusion (baseline), post-occlusion, and post-release (reperfusion)—and assessed for reproducibility and discriminability. ICGV was used for confirmation of occlusion.

#### Femoral artery imaging

To determine if real -time images could be obtained under basal conditions as well as during and after reversible occlusion of a significant artery, a rat femoral artery model was employed. Rats (n = 5) were positioned in supine position and the femoral artery and vein were exposed. After disinfection of the skin, a linear oblique incision, superficial to the inguinal ligament, on the right side of the abdomen was performed with consequent exposure of the region of interest. Real time images and raw data were acquired under basal conditions, during reversible femoral artery occlusion with a temporary clip, and during reperfusion after clip removal. Images under basal conditions were also taken for comparison after the administration, via tail injection, of 0.5 mg/kg ICG, as previously described^5^. Total time of occlusion was long enough to allow multiple images and data acquisition, however it was generally less than two minutes. Immediately after the clip removal, new images and data were acquired to detect eventual changes in blood flow during reperfusion.

#### Middle Cerebral Artery Occlusion (MCAO)

To more closely mimic the clinical scenario in which intraoperative imaging of blood flow could potentially add value, a model of rat middle cerebral artery occlusion was used. Rats (n = 5) were positioned in prone position and a curvilinear incision from the frontal midline to the left anterior peri-auricular region was performed. The temporalis muscle was incised and dissected following the skin flap thus exposing both the left fronto-parietal skull, in order to have access to the left paramedian fronto-parietal bone to perform the cortical window craniectomy (see below for the description of the exposure of the cortical window), and the temporal bone over the MCA. The temporalis bone was carefully drilled thus exposing the underlying dura. This was then carefully incised to have surgical access to the MCA. LSCI and ICG-VA images of the MCA were acquired under basal conditions and immediately (1–5 min) after cauterization.

#### Cortical window imaging

Cortical window imaging of a rodent MCA was utilized to determine accuracy of imaging at basal conditions and to assess the deficit and insult following cauterization and occlusion of a significant non-superficial blood vessel in the brain. After exposing the MCA and before proceeding with cauterization of the vessel as previously described, a small left fronto-parietal craniectomy (5 mm ×5 mm) was performed, thus exposing the underlying left parietal lobe. Real -time images and raw data for retrospective analyses were acquired under basal conditions, two hours after cauterization and twenty-four hours after occlusion.

#### Rabbit brain imaging

A rabbit model was employed to assess the capability of this system to image vessels within the brain parenchyma during an invasive cerebrovascular procedure. Another advantage of this model was that it allowed us to demonstrate successful imaging of real-time blood flow changes in differently sized brains and vessels. The rabbits (n = 3) were positioned in prone position. A cannula was placed in the auricular vein. After disinfection of the skin, a linear midline incision and a 20 × 20 mm craniectomy was performed thus displaying the cortical brain surface bilaterally. Real -time images and raw data were acquired under basal conditions, during irreversible, partial, and complete occlusion, through electro-cauterization, and after the occlusion. The partial cauterization was obtained by a very quick low voltage application of the cautery-tool on the vessel, whereas the complete cauterization through a longer higher voltage cautery. At each stage, real-time images and data for retrospective analyses were acquired. Under basal conditions and after cauterization, images were also acquired after the administration of 0.5 mg/kg of ICG, via cannula. According to protocol, the animals were euthanized after the procedure.

### Histological analysis

#### TTC staining

A common method to stain brain sections is 2,3,5-triphenyltetrazolium chloride (TTC). This technique was first introduced in 1984 and then further developed^58^. Briefly, TTC is a water-soluble tetrazolium salt with a white appearance. TTC is the substrate of cellular dehydrogenases of which the mitochondria are replete. When TTC enters the cells, it is reduced to an insoluble red compound called formazan giving viable cells a characteristic red color, that helps discriminate them from dead tissue which retains its original white hue. As previously described, TTC is dissolved in saline solution at a concentration of 2% and this solution is warmed to 37 °C. Twenty-four hours after the MCAO, after the image and data acquisition of the cortical window vessels, the rat brains were harvested, sliced on a coronal plane at intervals of 2 mm and dropped into the pre-warmed solution. After 30 minutes at 37 °C, the slices were transferred to formalin, images were taken, and the total and ischemic areas were calculated.

#### Infarct area measurements

As previously described^59^, all TTC slices were photographed and stored as TIFFs. Image J (Image J, Bethesda, MD) software was used to measure non-ischemic and ischemic infarct areas in select slices 2 mm apart, and aggregated to compute the mean total and mean ischemic area for each animal.

## Supplementary information

Supplementary Materials.
