# Supplementary material for: Intraoperative Laser Speckle Contrast Imaging For Real-Time Visualization of Cerebral Blood Flow in Cerebrovascular Surgery: Results From Pre-Clinical Studies
Source: Sci Rep. 2020 May 6;10:7614. doi: 10.1038/s41598-020-64492-5 (PMC7203106; doi:10.1038/s41598-020-64492-5)
Supplement: Supplementary file 1 — Supplementary Materials. [file 41598_2020_64492_MOESM1_ESM.pdf]

INTRAOPERATIVE LASER SPECKLE CONTRAST IMAGING FOR REAL-TIME VISUALIZATION OF CEREBRAL BLOOD FLOW IN CEREBROVASCULAR SURGERY: RESULTS FROM PRE-CLINICAL STUDIES

Antonella Mangraviti[*,1] Francesco Volpin[*,1] Jaepyeong Cha,[2] Samantha I. Cunningham,[2] Karan Raje,[2] M. Jason Brooke,[2] Henry Brem,[1,3,4,5] Alessandro Olivi,[1,3,6] Judy Huang,[1] Betty M. Tyler,[1**] and Abhishek Rege[2]

[1] Department of Neurosurgery, Johns Hopkins University, Baltimore, MD;

[2] Vasoptic Medical Inc., Baltimore, MD;

[3] Department of Oncology, Johns Hopkins University, Baltimore, MD;

[4] Department of Ophthalmology, Johns Hopkins University, Baltimore, MD;

[5] Department of Biomedical Engineering, Johns Hopkins University, Baltimore, MD;

[6] Department of Neurosurgery, Catholic University *School* of Medicine, Rome, Italy

[*]These authors contributed equally to this work

****Corresponding Author**: Betty Tyler (btyler@jhmi.edu)

SUPPLEMENTARY MATERIALS

Imaging process

For each stack of acquired speckle image frames, the speckle contrast (K), defined as the ratio of standard deviation of pixel intensities to the mean pixel intensity within a spatio-temporal neighborhood of pixels around every pixel P_0 (Eq. S1), was computed.

$$K(P_0) = \sigma_{\mathbb{N}(P_0)} / \mu_{\mathbb{N}(P_0)} \qquad \ldots\ldots \qquad \text{Eq. S1}$$

where $\sigma_{\mathbb{N}(P_0)}$ and $\mu_{\mathbb{N}(P_0)}$ are the standard deviation and mean, respectively, in the intensity of all pixels on a defined local neighborhood $\mathbb{N}(P_0)$. Traditionally, $K(P_0)$ values are calculated such that $\mathbb{N}(P_0)$ is chosen exclusively in a single image frame, that is, exclusively in the spatial domain;[53] however, this strategy compromises the spatial resolution of the estimated blood flow information. Preserving the spatial resolution is possible by calculating $K(P_0)$ values for every pixel across the stack of image frames, that is, exclusively in the temporal domain.[54] However, such a strategy would require at least 40 frames for a robust estimation of blood flow leading to significant temporal averaging and increased latency of the video output. Therefore, to optimize the spatio-temporal resolution and image acquisition times, the SurgeON System calculates speckle contrast using a spatio-temporal pixel-neighborhood of 5 pixels x 5 pixels (spatial window, S) x 5 frames (temporal window, N) around every pixel P_0 (Eq. S2) for contrast calculation: [55]

$$\mathbb{N}(P_0) = \left\{ \begin{matrix} P(x, y, n)\ s.t. \\ \|(x, y) - (x_0, y_0)\| \leq\ S\ px \\ |n - n_0| \leq N\ frames \end{matrix} \right\} \qquad \ldots\ldots \qquad \text{Eq. S2}$$

The correlation time of intensity fluctuations observed in speckle dynamics over the exposure time of the camera, is known to be inversely proportional to the velocity of the moving scatterers, therefore, providing a means to estimate a blood flow velocity index (BFVI). BFVI can be mathematically computed from K at every pixel at a given exposure time T of the camera using Eq. S3.[56,57]

$$K^2 = \frac{1}{T\times BFVI}\left\{2 - \frac{[1-exp(-2T\times BFVI)]}{T\times BFVI}\right\} \qquad \ldots\ldots \qquad \text{Eq. S3}$$

The SurgeON displays a pseudo-color representation of BFVI values obtained through the use of look up tables.

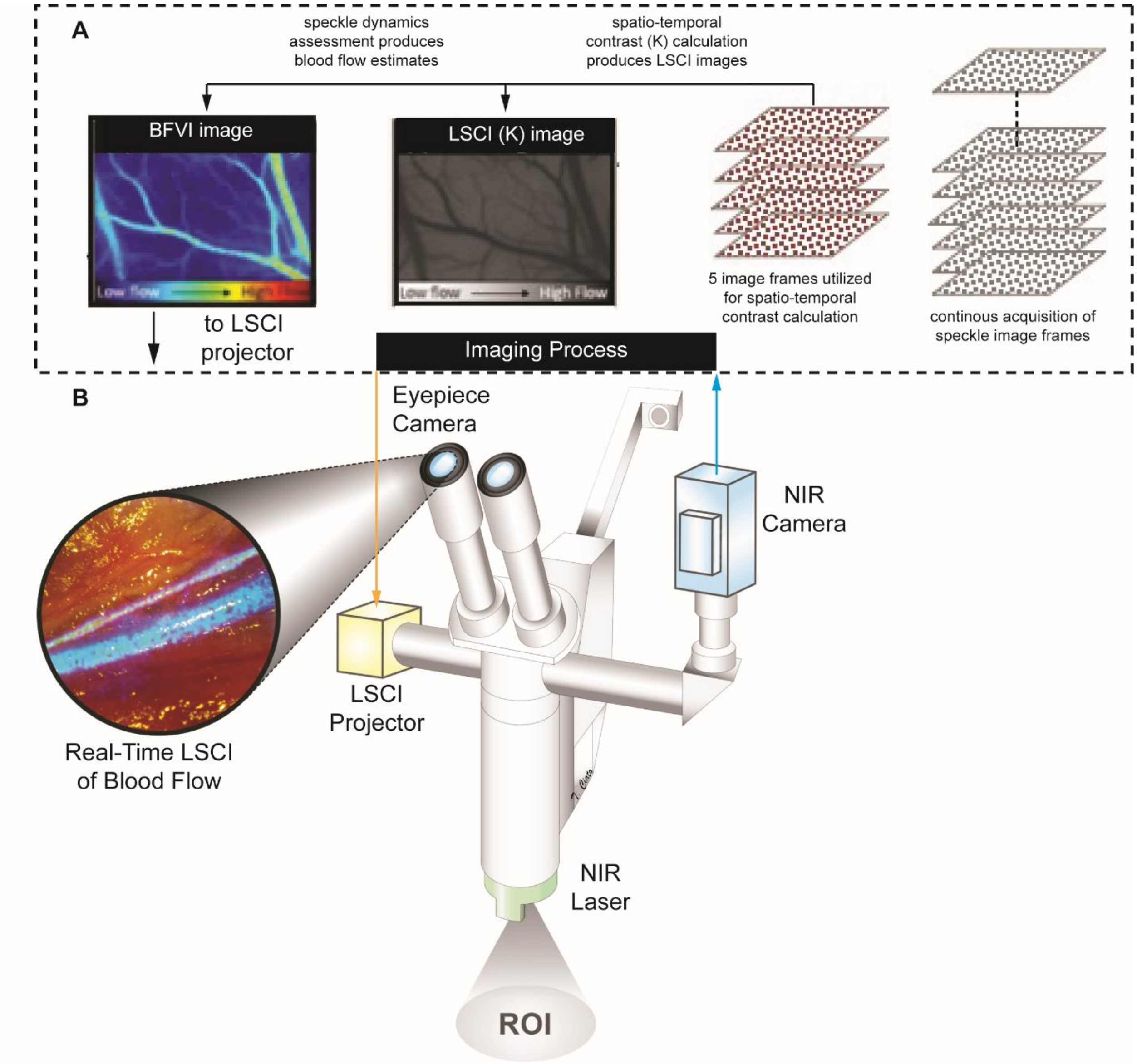

Supplementary Fig S1: Schematic representation of the SurgeON imaging process (A) and modified microscope (B).

SurgeON System calibration

We set up an *in vitro* microfluidic system to test the technical specifications of the SurgeON. The flow was varied across a wide range (4 mm/s – 160 mm/s) to cover the practical range of velocities encountered *in-vivo*. Imaging was done as per the protocol described below and repeated at different exposures (0.5 ms through 8 ms). Care was taken to not disturb or move the setup when changing the exposure times. A syringe filled with rat blood was fitted into a syringe pump (PHD2000, Harvard Apparatus, MA) and connected to a polyethylene tube (internal diameter 1/32”). Blood was infused into the tube at different rates, in accordance with practically observed vascular velocities. Once the flow was steady (about 1 minute after the flow was started), the tube was imaged using the LSCI modality of the SurgeON System prototype. At each flow rate, LSCI was carried out at multiple exposure times of the camera for calibration.

As reported, the coefficient of determination (R-squared) for the logarithmic best fit at each exposure time is greater than 0.94 over all the physiologically relevant flow rates. The exposure time can be adjusted based on expected flow rates in the region of interest.

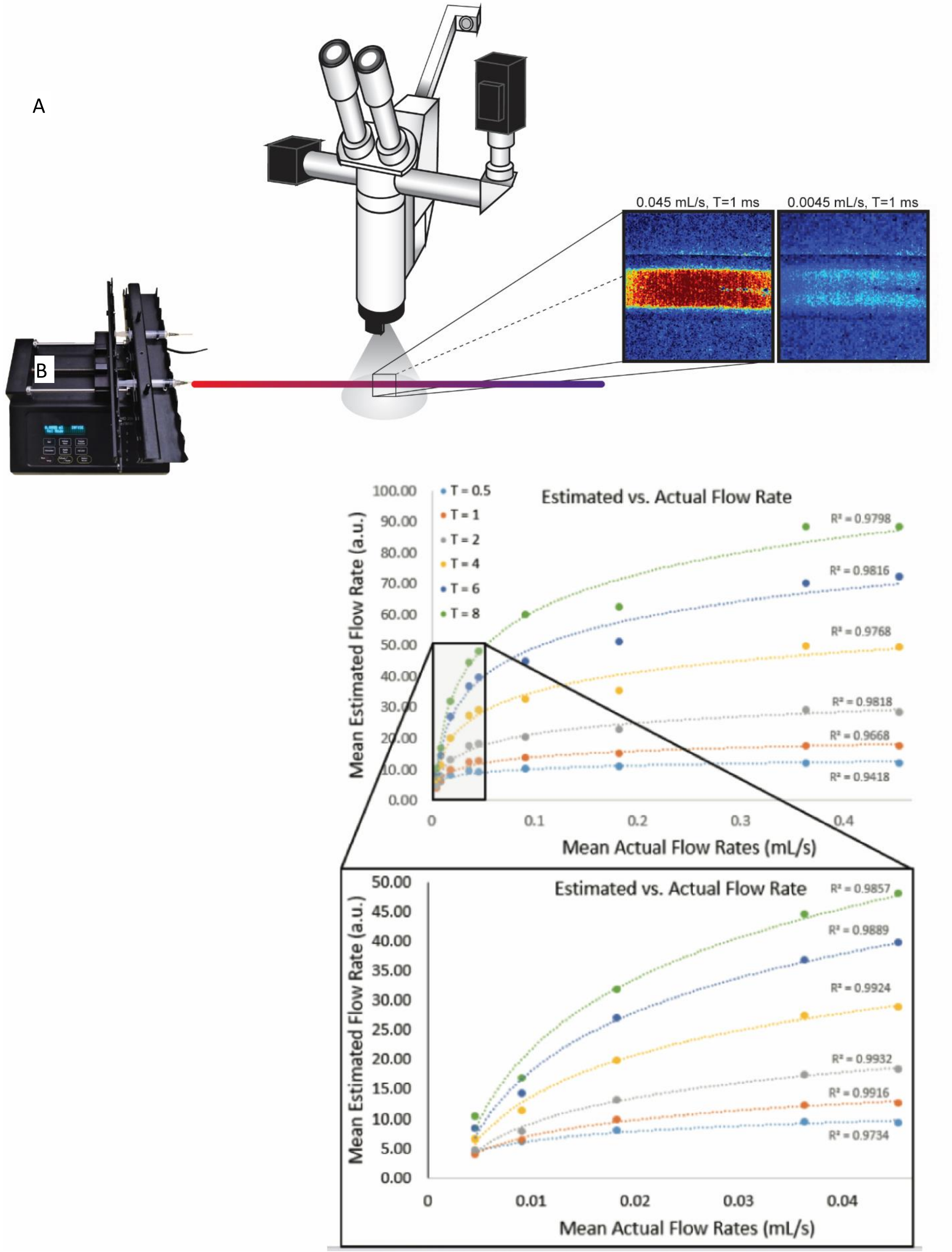
A
B
0.045 mL/s, T=1 ms
0.0045 mL/s, T=1 ms
Estimated vs. Actual Flow Rate
T = 0.5
T = 1
T = 2
T = 4
T = 6
T = 8
R² = 0.9798
R² = 0.9816
R² = 0.9768
R² = 0.9818
R² = 0.9668
R² = 0.9418
Mean Estimated Flow Rate (a.u.)
Mean Actual Flow Rates (mL/s)
Estimated vs. Actual Flow Rate
R² = 0.9857
R² = 0.9889
R² = 0.9924
R² = 0.9932
R² = 0.9916
R² = 0.9734
Mean Estimated Flow Rate (a.u.)
Mean Actual Flow Rates (mL/s)

Supplementary Fig S2: Calibration of the SurgeON System Prototype

Fig. S2A shows the experimental setup with two example BFVI images of blood flowing at rates of 0.0045 mL/s (right) versus 0.045 mL/s (left) using an exposure time (T) of 1ms. Fig. S2B reports the measured BFVI values as a function of the actual flow rate (set by the pump) and the exposure time (T) of the camera.

Rats	Relative BFVI Pre-cautery	Relative BFVI 2hs Post-cautery	Relative BFVI 24hs Post-cautery	% Infarcted Area
#1	36.53 ± 12.53	17.7 ± 5.16	33.26 ± 9.86	3.1%
#2	44.93 ± 13.56	27.11 ± 7.61	25.29 ± 4.36	6%
#3	95.09 ± 13.91	34.93 ± 3.65	39.84 ± 4.87	3.4%
#4	44.75 ± 6.57	13.85 ± 5.09	11.32 ± 3.1	6.9%
#5	44.37 ± 8.56	39.85 ± 13.36	33.14 ± 12.35	4.7%

Supplementary Table 1: BFVI in cortical vessels and percentage of infarcted area

The table show that the CBF (1/tauc) values pre and post cautery in the cortical vessel and the % of the infarcted area at 24 hours post MCAO of all five rats. The % of the infarcted area is significantly higher the rats #2, #4, and #5 compared to rats #1 and #2, and it correlates with relative lower BFVI at 24hours post-cautery in these rats.

Statistical Analyses

GraphPad Prism Software (Version 6.0, GraphPad Software, San Diego, CA), Excel (Microsoft, Redmond, WA), and MATLAB (Mathworks, Natick, MA) were used for all statistical analyses. Graphs represent the mean ± standard deviation of the relative blood flow velocity indices (BFVI, 1/tauc values) acquired: in 5 data sets per condition, across 5 different rats, in the femoral/vein CBF analysis; on 3 vessels per rat in a total of 5 rats, in the brain cortical vessel cautery experiment; and on 3 vessels in 3 rabbits in the cauterization experiment. A Student's t-test was used to assess differences in relative BFVI between conditions for each experiment. The analysis of BFVI changes before and after brain MCAO at different time points was carried out using one-way ANOVA with Bonferroni/Tukey post-tests, depending on the type of distribution of the data (following D'agostino test). Intrasession coefficients of variation (CV) were calculated as the ratio of standard deviation of BFVI measurements to the mean BFVI measurement, and expressed as a percent value.
